# Erythromycin for Promoting the Postpyloric Placement of Feeding Tubes: A Systematic Review and Meta-Analysis

**DOI:** 10.1155/2018/1671483

**Published:** 2018-04-03

**Authors:** Qing-Jun Jiang, Cai-Feng Jiang, Qi-Tong Chen, Jian Shi, Bin Shi

**Affiliations:** ^1^Department of Gastroenterology, Changzheng Hospital, Second Military Medical University, Shanghai 200003, China; ^2^Department of Emergency Medicine, Changzheng Hospital, Second Military Medical University, Shanghai 200003, China

## Abstract

**Background:**

Critically ill patients can benefit from enteral nutrition with postpyloric feeding tubes, but the low success rate limits its wide use. Erythromycin could elevate the success rate of tube insertion, but its clinical efficiency still remains controversial.

**Methods:**

Included studies must be RCTs which assessed the success rate of postpyloric feeding tube insertion using erythromycin.

**Results:**

284 patients were enrolled in six studies. Meta-analysis showed that erythromycin significantly increases the rate of successful postpyloric feeding tube placement (RR 1.45, 95% CI (1.12, 1.86)) and did not increase the risk of adverse effects (RR 2.15, 95% CI (0.20, 22.82)). Subgroup analysis showed that unweighted feeding tubes (RR 1.47, 95% CI (1.03, 2.11)) could significantly increase the success rate. Country of study, intravenous route of erythromycin, and year of participant enrollment did not influence these results.

**Conclusions:**

Erythromycin significantly increases the success rate of postpyloric feeding tube placement. This suggests that erythromycin can be used as an auxiliary method to improve the success rate of bedside insertion.

## 1. Background

Successful and early administration of enteral nutrition is important for critically ill patients [1–3]. It can boost immune function, decrease infectious complications, and improve wound healing. However, 50%–60% of critically ill patients suffer from gastroparesis [4]. Due to delayed gastric emptying, the success rate is low in feeding tube insertion and inadequate nutrition, which could even cause gastroesophageal reflux [5]. Once the gastroesophageal reflux occurs, it may cause pulmonary aspiration, pneumonia, and sepsis, which further impacts on mortality.

Postpyloric feeding tube can reduce the risk of complications, because it delivers nutrient directly to the duodenum, which is just like a protective barrier against reflux, instead of the stomach. Usually, there are three methods to place postpyloric tube: bedside blind insertion, insertion under X-ray, and insertion with endoscopy. Because the bedside insertion can be performed conveniently and can also reduce the pain of insertion, it is especially suitable for critically ill patients. Unfortunately, a study reported that only 53.5% of 932 blind postpyloric tube placement attempts succeeded, which means that blind insertion had its disadvantage [6].

Erythromycin is not only an antibiotic, but also a gastric prokinetic drug. It is a motilin receptor agonist which can promote motilin secretion and thereby enhance the gastric emptying [7]. Previous studies showed that erythromycin could increase the success rate of postpyloric tube insertion [8–11], but its clinical efficiency still remains controversial. The purpose of this meta-analysis was to evaluate the success rate and complications of erythromycin in postpyloric tube insertion.

## 2. Methods

### 2.1. Search Strategy

Relevant articles were retrieved from Medline, Embase, Web of Science, China national knowledge internet, ChinaInfo, and the Cochrane controlled trials registered from update to January 2018. The following words “erythromycin, gastrointestinal motility, enteral nutrition, nasogastric feeding, post-pyloric feeding tubes” were used as retrieval words. The retrieval language was not limited to English. The references from articles were also used.

### 2.2. Study Selection

Included studies must meet the following criteria:

1. Study design: randomized controlled trial (RCT)
2. Population: critically ill patients included adults who needed enteral nutrition through postpyloric feeding tubes
3. Intervention: the patients in the experimental group given with erythromycin before inserting the postpyloric feeding tubes
4. Control: the patients in the control group given either no intervention or the same dosage of normal saline
5. Outcomes: success rate of postpyloric feeding tube insertion

### 2.3. Data Extraction

Two reviewers extracted the following data: first author, year of publication, characteristics of patients, study design, feeding tube characteristics, data of interventions, outcomes, risk of bias, and adverse effects. Two reviewers resolve disagreements by discussion and consensus.

### 2.4. Assessment of Risk of Bias

We used the Cochrane risk of bias to assess trials for risk of bias [12]. We assessed the following domains separately for each of the included studies as “low risk of bias,” “high risk of bias,” and “unclear” when the risk of bias was uncertain or unknown:

1. Adequate sequence generation
2. Allocation concealment
3. Blinding of participants and personnel
4. Blinding of outcome assessment
5. Incomplete outcome data
6. Selective outcome reporting
7. Other bias

The overall risks of bias for the included studies were categorized as low if the risk of bias is low in all domains, unclear if the risk of bias was unclear in at least one domain and with no high risk of bias domain, or high if the risk of bias was high in at least one domain. Any disagreement was resolved by consensus.

### 2.5. Statistical Analysis

We estimate the pooled risk ratio (RR) with 95% confidence intervals (CIs) for dichotomous outcomes by using Review Manager 5.3 (Cochrane IMS, Oxford, UK). The pooled RRs were calculated by the Mantel-Haenszel estimator, and WMDs were estimated by the inverse variance approach. Statistical heterogeneity was assessed by calculation of standard chi^2^ test and *I*^2^ statistics. We defined chi^2^ < 0.1 or *I*^2^ > 50% as significant heterogeneity. Subgroup analysis was performed to explore whether certain factors influenced clinical effect. The certain factors included tip of the feeding tubes, route of administration, age, country, and dose.

## 3. Results

### 3.1. Study Identification and Selection

**Figure 1:**
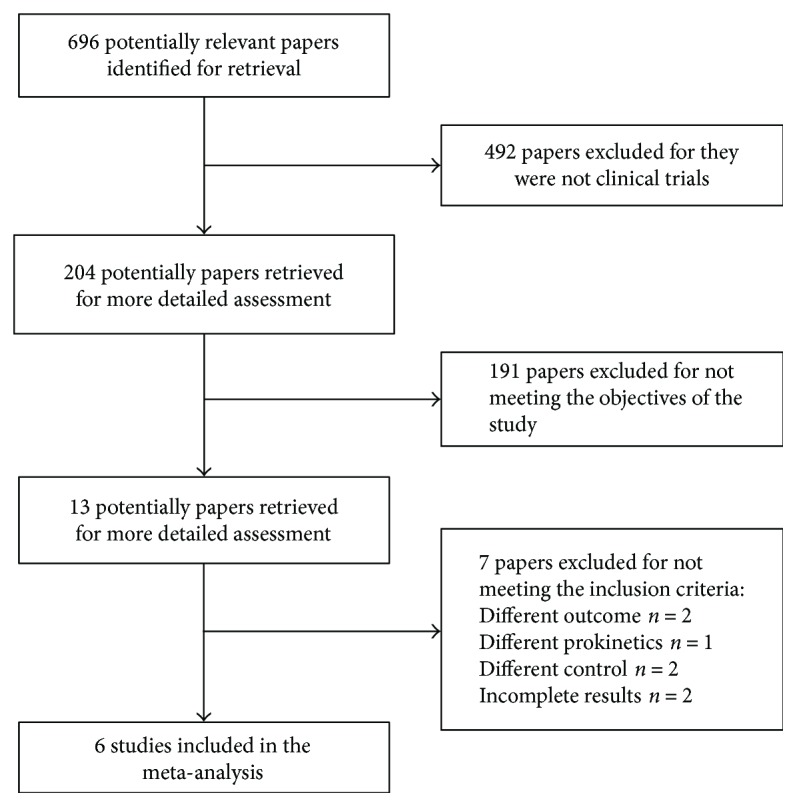
Flow chart of the selection of studies.

The search strategy identified 696 potentially relevant papers. Of these, 492 papers were excluded for they were not clinical trials. 191 papers did not meet the inclusion criteria. We assessed 13 papers in detail, of which 7 papers were excluded for the following reasons: different outcomes (*n* = 2), different prokinetics (*n* = 1), different controls (*n* = 2), and incomplete results (*n* = 2). Finally, six studies [8–11, 13, 14] met the inclusion criteria and were included into the meta-analysis, and they were all prospective randomized controlled trials (Figure 1). In total, 284 patients were enrolled, of which 144 were treated with erythromycin and 140 with placebo. Of the six studies, 169 postpyloric feeding tubes were successfully inserted.

The characteristics of the six studies are shown in Table 1. Three clinical trials were carried out in the United States [8–11, 13, 14], two in China [13, 14], and one in Netherlands [11]. Patients received erythromycin with intravenous route in all six studies. The feeding tube characteristics were listed in Table 2. Two types (weighted tip, unweighted tip) of enteral feeding tubes were involved in six studies. Three studies used feeding tubes with a weighted tip [8, 9, 13]; one of which compared the weighted tip feeding tubes with unweighted tip feeding tubes [9]. Three studies used the feeding tubes with a Hydromer coating tip, which lubricated it after submersion in water [10, 11, 14].

### 3.2. Risk of Bias

**Figure 2:**
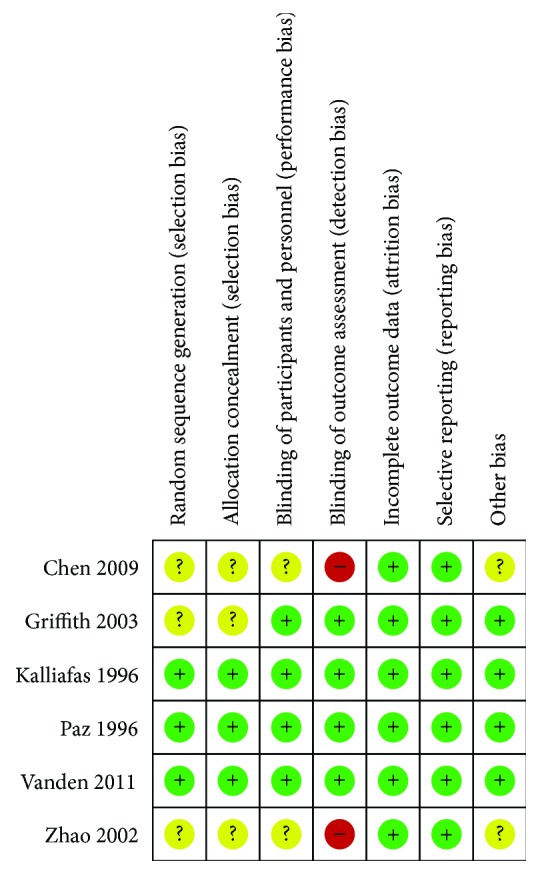
Risk of bias summary: review authors' judgements about each risk of bias item for each included study. Green circles indicate low risk of bias, yellow circles unclear risk of bias, and red circles high risk of bias.

The risks of bias of the included studies were summarized in Figure 2. Cochrane risk-of-bias tool was used to judge included studies. Four studies were at the low risk of bias [8, 9, 11, 15], two studies were assessed with the high risk of bias because there were no descriptions of double-blind methods [13, 14], and we were not able to assess the risk of bias in two studies due to lack of information [10, 16].

### 3.3. Main Outcomes

**Figure 3:**
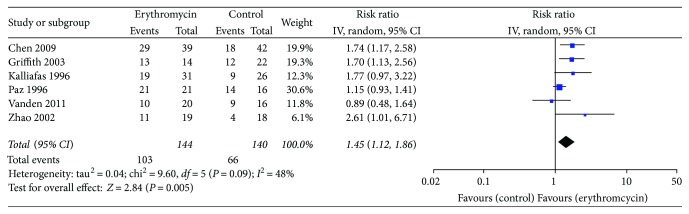
Successful postpyloric feeding tube placement. Forest plot includes pooled estimates for randomized controlled trials comparing erythromycin to placebo for successful insertion of postpyloric tube outcome.

**Figure 4:**
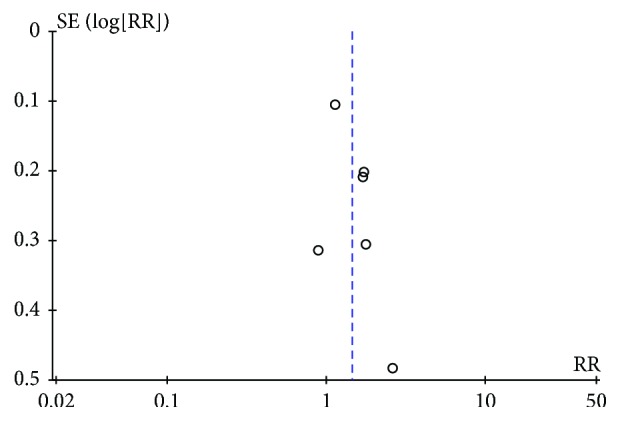
The funnel plots for successful insertion of postpyloric tube outcome.

Regardless of the dose, frequency, and duration of erythromycin, success of postpyloric feeding tube placement was 103 out of 144 participants (71.5%) using erythromycin compared with 66 out of 140 (47.1%) in the control group. Meta-analysis showed that erythromycin significantly elevated the rate of successful postpyloric feeding tube placement (RR 1.45, 95% CI (1.12, 1.86); *P* = 0.005). In this comparison, there was statistically significant heterogeneity (chi^2^ = 9.60, df = 5, *P* = 0.09, *I*^2^ = 48%) (Figure 3). The funnel plots for clinical events showed slight asymmetry, suggesting the possibility of publication bias (Figure 4).

### 3.4. Adverse Effects

**Figure 5:**
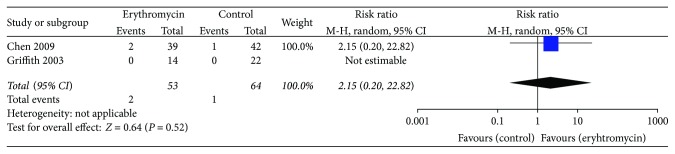
Meta-analysis of adverse effects.

There were two studies reporting adverse effects of erythromycin. Compared with placebo, erythromycin did not increase the risk of adverse effects such as vomiting, loose stool, and phlebitis (RR 2.15, 95% CI (0.20, 22.82); *P* = 0.52, *I*^2^ = 0%) (Figure 5). Van den Bosch et al. [11] noted that adverse events included pain (*n* = 1), nausea (*n* = 2), and vomiting (*n* = 1), but did not report which group the adverse effects occurred in.

### 3.5. Subgroup Analysis and Sensitivity Analysis

#### 3.5.1. Subgroup Analysis of Doses

The results of the meta-analysis are given in Table 3. All studies assessed the success rate related to erythromycin. When we removed the oral medication study and compared erythromycin 200–250 mg with placebo [8, 9, 11, 13, 14], the success rate of the erythromycin group was significantly higher than that of the control group (RR 1.42, 95% CI (1.00, 2.03); *P* = 0.05, *I*^2^ = 64%).

**Figure 6:**
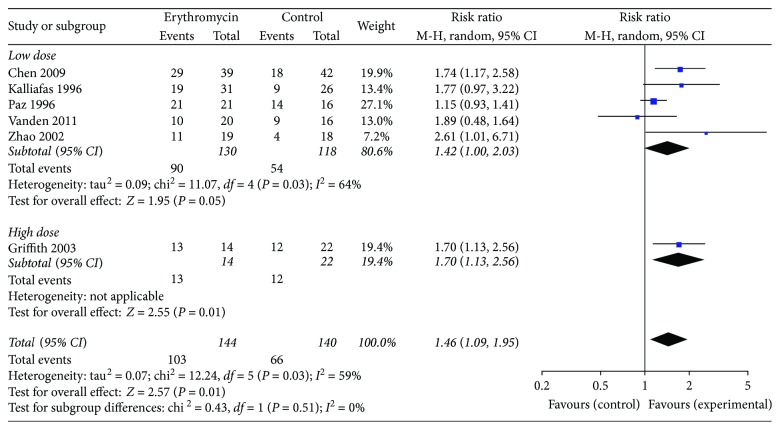
Different doses of erythromycin versus placebo for successful insertion of postpyloric tube outcome.

When patients receive 500 mg erythromycin compared with control [10], the success rate of the erythromycin group (92.8%) was higher than that of the control group (54.5%), and the success rate of the erythromycin group was significantly higher than that of the control group (RR 1.70, 95% CI (1.13, 2.56); *P* = 0.01) (Figure 6).

#### 3.5.2. Subgroup Analysis of Enteral Feeding Tubes

**Figure 7:**
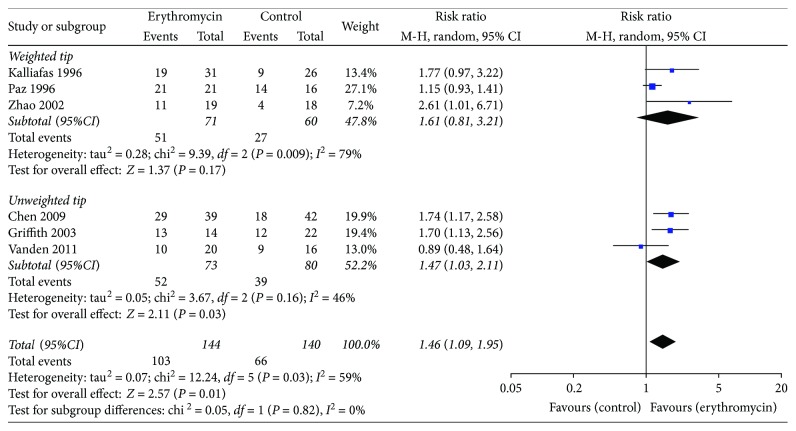
Different types of enteral feeding tubes for successful insertion of postpyloric tube outcome.

There were two types of enteral feeding tubes, one with weighted tip and the other with Hydromer coating tip. In the studies using the weighted tip feeding tubes, there was no difference between the two groups (RR 1.61, 95% CI (0.81, 3.21); *P* = 0.17, *I*^2^ = 79%). However, in the studies using the unweighted feeding tubes, the success rate of the erythromycin group was significantly higher than that of the control group (RR 1.47, 95% CI (1.03, 2.11); *P* = 0.03, *I*^2^ = 45%) (Figure 7).

#### 3.5.3. Subgroup Analysis of Countries

**Figure 8:**
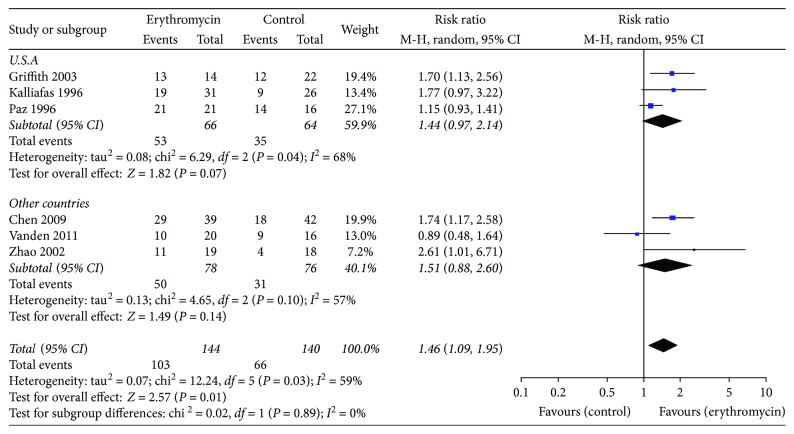
Different countries using enteral feeding tubes for successful insertion of postpyloric tube outcome.

In the subgroup analysis, trials were aggregated according to the country of study. We did not find the statistically significant difference between the erythromycin group and the control group, neither carried out in the United States (RR 1.31, 95% CI (0.91, 1.87); *P* = 0.14, *I*^2^ = 71%) nor in the other countries (RR 1.51, 95% CI (0.88, 2.60); *P* = 0.14, *I*^2^ = 57%) (Figure 8).

## 4. Discussion

Critically ill patients can benefit from enteral nutrition with postpyloric feeding tubes, but the low success rate limits the wide use of postpyloric feeding tubes. A previous study showed that postpyloric tubes had higher success rate (50%) in patients with normal gastric emptying, but lower success rate (28.6%) in patients with delayed gastric emptying [17]. The authors suggested that impaired gastric motility was an important factor leading to a low success rate.

Erythromycin, the first macrolide antibiotic used in clinical practice, was discovered in 1952 [18]. Erythromycin could stimulate gastrointestinal motility because it acts as a motilin receptor agonist in the gut and gallbladder stimulating enteric nerves and smooth muscle and triggering a phase of the migrating myoelectric complex [18]. In 1990, Janssens et al. used erythromycin as a gastrointestinal prokinetic agent to improve impaired gastric emptying in patients with severe diabetic gastroparesis [19]. Thereafter, many studies had been carried out in a wide variety of patient populations and disorders, including gastroesophageal reflux [20] and diabetic gastroparesis [21]. In 1994, the first randomized controlled trial by Stern et al. showed that erythromycin could significantly increase the success rate of placing postpyloric feeding tubes [16]. This paper was excluded, because the administration route was oral and the further detailed regimen was not reported.

A previous study demonstrated that erythromycin was a prokinetic agent and markedly stimulated antral contractions in a dose-dependent manner in critical care patients [22]. The two different types of motilin receptors may cause the different effects of high and low doses of erythromycin. Researchers proposed that low doses (1–3 mg/kg) of erythromycin stimulated the neuroreceptor and then triggered the migrating motor complex (MMC), while high doses (10 mg/kg) might stimulate the muscle receptors and then triggered antral contractions, inhibiting MMCs [23]. Some studies suggested that intravenous dose of erythromycin (200–250 mg) could increase postpyloric migration of feeding tubes [23]. Besides, several studies showed higher postpyloric insertion success rates with erythromycin (400–500 mg) [10, 16].

Before 2000, clinicians thought that tube placement with a weighted tip could increase the success rate [8, 9]. However, there were no statistically significant differences between erythromycin and placebo when using the weighted enteral tubes. Not surprisingly, recent studies [10, 14] used the unweighted enteral tubes instead of weighted enteral tubes. The tip of an unweighted enteral tube has a Hydromer coating, which lubricates it after submersion in water. Our results showed that postpyloric insertion success rate of the erythromycin group was higher than that of the control group using the unweighted enteral tubes. Subgroup analysis and sensitivity analysis found that some factors, such as country, intravenous route, and adult participants, did not change the results.

Our study was the first meta-analysis which focused on the effect of erythromycin on postpyloric feeding tube insertion [24]. Some factors, like the dose of erythromycin and the type of enteral tubes, might affect the success rate. Despite these findings, this meta-analysis had several limitations. We included 6 randomized controlled trials, but only half of them were of high quality. In addition, some studies did not report some important outcomes, such as adverse events. Furthermore, the included studies varied in selection criteria, treatment protocols, type of enteral tubes, and the time to assess tube position after tube insertion. Finally, this review might contain selection and publication bias.

## 5. Conclusions

Erythromycin significantly increases the success rate of postpyloric feeding tube placement. An unweighted feeding tube may successfully achieve postpyloric placement for early initiation of nutrition in critically ill patients. This suggests that erythromycin can be used as an auxiliary method to improve the success rate of bedside insertion.

## Figures and Tables

**Table 1:** Baseline characteristics of the included studies.

Author	Country	Subjects	Patients (*n*) T/C	Age (yr) T/C	M/F	Intervention groups T/C	Method for judging feeding tube	Outcomes
Griffith 2003	USA	Surgical critically ill patients requiring enteral nutrition Age: ≥18 years	14/22	54.6/59.7	25/11	Erythromycin 500 mg IV single dose versus saline placebo	An abdominal X-ray was obtained within 1 hr of the procedure	Successful postpyloric feeding tube insertion
Kalliafas 1996	USA	Surgical critically ill patients requiring enteral nutrition Age: ≥18 years	31/26	54.7/57.3	30/27	Erythromycin 200 mg IV single dose versus saline placebo	An abdominal X-ray was obtained within 30 min of the procedure	Successful postpyloric feeding tube insertion
Paz 1996	USA	Critically ill patients requiring enteral nutrition Age: 22–85 years	21/16	58.9/68.4	36/27	Erythromycin 200 mg IV single dose versus saline placebo	A chest radiograph was obtained 30 min after tube placement	(1) Successful postpyloric feeding tube insertion (2) Number of attempts prior to successful tube placement
Van den 2011	Netherlands	Acute pancreatitis patients requiring jejunal enteral nutrition (exclude ICU) Age: 18–80 years	22/18	49.0/52.0	17/23	Erythromycin 250 mg IV four dose versus saline placebo	An abdominal X-ray was obtained at 24/48 hr of the procedure	Successful postpyloric feeding tube insertion
Zhao 2002	China	Critically ill patients requiring enteral nutrition Age: 26–85 years	19/18	58.0/60.0	25/12	Erythromycin 250 mg IV single dose versus saline placebo	An abdominal X-ray was obtained within 3 hr of the procedure	Successful postpyloric feeding tube insertion
Chen 2009	China	Critically ill patients requiring enteral nutrition Age: ≥18 years	39/42	72.1/65.9	50/31	Erythromycin 250 mg IV two dose versus saline placebo	An abdominal X-ray was obtained after 24 hr of the procedure	Successful postpyloric feeding tube insertion

**Table 2:** Feeding tube characteristics of the included studies.

Study author	Tube type	Tube material	Tube diameter (mm)	Tube length (cm)	Tube tip
Griffith 2003	Corpak, Wheeling, IL	NR	3.3	109	Hydromer coating
Kalliafas 1996	Corpak, Wheeling, IL	NR	NR	109	Weighted tip
Paz 1996	Flexiflo	NR	NR	45	Weighted tip
Vanden 2011	Flocare Bengmark	Polyurethane	3.3	145	Hydromer coating
Zhao 2002	Corpak, Wheeling, IL	NR	NR	NR	Weighted tip
Chen 2009	Flocare Bengmark	Polyurethane	3.3	145	Hydromer coating

**Table 3:** Erythromycin versus placebo or no intervention for postpyloric placement of enteral feeding tubes.

	Number of studies	Number of participants	Rate (%) (erythromycin/control)	RR (95% CI)	*P*
Total	6	284	71.5/47.1	1.46 (1.09, 1.95)	0.01
Erythromycin 200–250 mg	5	248	69.2/45.7	1.42 (1.00, 2.03)	0.05
Erythromycin 500 mg	1	36	92.8/54.5	1.70 (1.13, 2.56)	0.01
